# Experimental evidence that immune trypanolysis using the LiTat 1.3 and LiTat 1.5 variant antigen types is not specific to *Trypanosoma brucei gambiense* in pigs[Fn FN1]

**DOI:** 10.1051/parasite/2022063

**Published:** 2022-12-21

**Authors:** Kadidiata Ilboudo, Robert Eustache Hounyeme, Jacques Kabore, Alain Boulangé, Geoffrey Gimonneau, Ernest Salou, Adrien Gaston Marie Belem, Veerle Lejon, Charlie Franck Alfred Compaoré, Bruno Bucheton, Mathurin Koffi, Philippe Solano, David Berthier, Sophie Thevenon, Vincent Jamonneau

**Affiliations:** 1 Unité de Recherche sur les Maladies à Vecteurs et Biodiversité, Centre International de Recherche-Développement sur l’Élevage en Zone Subhumide 01 BP 454 Bobo-Dioulasso 01 Burkina Faso; 2 Unité de Formation et de Recherche Sciences et Techniques, Université Nazi Boni 01 BP 1091 Bobo-Dioulasso Burkina-Faso; 3 Unité de Recherche « Trypanosomoses », Institut Pierre Richet 01 BP 1500 Bouaké Côte d’Ivoire; 4 Université de Montpellier, CIRAD, IRD, Intertryp F-34398 Montpellier France; 5 Laboratoire National d’Élevage et de Recherches Vétérinaires, Service de Bio-Écologie et Pathologies Parasitaires BP 2057 Dakar – Hann Sénégal; 6 Institut du Développement Rural, Université Nazi Boni 01 BP 1091 Bobo-Dioulasso 01 Burkina Faso; 7 Laboratoire de Biodiversité et Gestion des Écosystèmes Tropicaux, Unité de Recherche en Génétique et Épidémiologie Moléculaire, UFR Environnement, Université Jean Lorougnon Guédé BP 150 Daloa Côte d’Ivoire; 8 CIRAD, UMR INTERTRYP F-34398 Montpellier France

**Keywords:** *Trypanosoma brucei*, Human African trypanosomiasis, Trypanolysis, Diagnosis, Experimental infection, Pig

## Abstract

In the context of the human African trypanosomiasis elimination process, reliable and accurate diagnostic tools are crucial for exploring the role of a potential animal reservoir of *Trypanosoma brucei gambiense*. The immune trypanolysis test (TL) using the variant antigen types (VAT) LiTat 1.3 and LiTat 1.5, described as a specific serological method to detect people infected by *T. b. gambiense*, seems to be a promising tool. However, its specificity was recently questioned during field animal surveys. The present study evaluates the performance of TL during experimental *T. b. brucei* infection in pigs. Eight infected pigs and four uninfected pigs were followed up with blood and plasma collection. Blood was used for parasitological investigation. TL was performed on the plasma with the LiTat 1.3, LiTat 1.5 and LiTat 1.6 VATs. All control pigs remained negative to parasitological investigation and TL. Trypanosomes were detected in all the infected pigs and the first detection was between 10 and 14 days post infection (dpi). TL results showed that infected pigs developed antibodies against the three VATs. The first antibody detections by TL occurred between 14 and 21 dpi for antibodies directed against LiTat 1.6, 21 and 168 dpi for antibodies directed against LiTat 1.5 and 70, and 182 dpi for antibodies directed against LiTat 1.3. This study highlights for the first time that TL using LiTat 1.3 and LiTat 1.5 VATs is not specific to *T. b. gambiense*. Development of specific diagnostic tools for the detection of *T. b. gambiense* infections in animals, especially in pigs, is still needed.

## Abstract (fr)

Dans le contexte d’élimination de la trypanosomiase humaine Africaine, des outils de diagnostic fiables et précis sont essentiels afin d’explorer le rôle d’un potentiel réservoir animal de *Trypanosoma brucei gambiense*. La trypanolyse (TL) qui utilise les types d’antigène variable (TAV) LiTat 1.3 et LiTat 1.5, et qui est décrite comme une méthode sérologique spécifique pour détecter les personnes infectées par *T. b. gambiense*, semble être un outil prometteur. Cependant, sa spécificité a été récemment remise en question lors d’enquêtes sur les animaux. La présente étude évalue la performance de ce test lors d’une infection expérimentale à *T. b. brucei* chez le porc. Huit porcs infectés et quatre porcs témoins non infectés ont été suivis avec des prélèvements de sang et de plasma. Le sang a été utilisé pour l’examen parasitologique. La TL a été réalisée sur les échantillons de plasma avec les TAV LiTat 1.3, LiTat 1.5 et LiTat 1.6. Tous les porcs témoins ont été négatifs en parasitologie et à la TL. Les trypanosomes ont été détectés sur tous les porcs infectés avec les premières détections entre 10 et 14 jours post-infection (jpi). Les résultats de la TL ont montré que les porcs infectés ont développé des anticorps contre les trois TAV. Les premiers anticorps détectés par la TL étaient dirigés contre le LiTat 1.6 entre 14 et 21 jpi, puis le LiTat 1.5 entre 21 et 168 jpi et enfin le LiTat 1.3 entre 70 et 182 jpi. Cette étude démontre pour la première fois que la TL basée sur les TAV LiTat 1.3 et LiTat 1.5 n’est pas spécifique de *T. b. gambiense*. Il est donc toujours nécessaire et urgent de développer un outil de diagnostic spécifique pour la détection des infections à *T. b. gambiense* chez les animaux, notamment chez les porcs.

## Introduction

African trypanosomes are parasites of the genus *Trypanosoma* that cause disease in both humans and animals. They are extracellular flagellate protozoa mainly transmitted by tsetse flies (genus *Glossina*). Within the *T. brucei* species, *T. b. gambiense* and *T. b. rhodesiense* are the pathogens of human African trypanosomiasis (HAT), also called sleeping sickness, in Western and Central Africa, and Eastern and Southern Africa, respectively [43]*. T. b. brucei,* alongside *T. congolense* and *T. vivax,* causes animal African trypanosomosis (AAT) or nagana in a wide range of domestic and wild animals in sub-Saharan Africa [10, 11, 40]. While AAT still causes considerable economic losses [1, 24], recent efforts to control HAT have drastically reduced the prevalence of the disease. The elimination as a public health problem of the *T. b. gambiense* HAT chronic form (g-HAT), causing 87% of HAT cases (1441 cases reported in 2019 and 2020), is being achieved, and the World Health Organization now targets the interruption of g-HAT transmission for 2030 [14].

However, the existence and the epidemiological role of a potential domestic and wild animal reservoir of *T. b. gambiense* may compromise this objective [3]. As mentioned in the WHO road map for neglected tropical diseases 2021–2030, the scientific community has been requested to elucidate this crucial question [43]. Recent studies conducted in Côte d’Ivoire showed that pigs often carry multiple trypanosome infections and highlighted the lack of tools to prove or exclude with certainty the presence of *T. b. gambiense* [30, 39]. The challenge is to distinguish between the subspecies *T. b. brucei* and *T. b. gambiense* that may be found in sympatry. These two sub-species are morphologically indistinguishable and the TgsGP1/2 PCR [34] targeting the TgsGP gene [2] and considered the only available tool specific of *T. b. gambiense* lacks sensitivity [6].

The immune trypanolysis (TL) based on the detection of antibodies against predominant or specific variant antigen types (VAT) [41] is increasingly used for the epidemiological surveillance of g-HAT [7, 8, 21, 23] and it was also recently applied to animal surveys conducted in Côte d’Ivoire. With positivity rates of up to 50% that were inconsistent with HAT prevalence in the studied hypo-endemic or historical foci, the *T. b. gambiense* specificity of VAT LiTat 1.3 and LiTat 1.5 was questioned [30, 39]. This specificity was also questioned with positive TL results observed with these two VAT in cattle in a *rhodesiense* HAT and AAT endemic region in Uganda [27]. The aim of this study was to determine the *T. b. gambiense* specificity of VAT LiTat 1.3 and LiTat 1.5 and the diagnostic performance of TL, during *T. b. brucei* experimental infection in pigs. We also included the VAT LiTat 1.6 already known to be common to *T. brucei* species.

## Material and methods

### Ethical statement

The study protocol was approved by the Comité d’Ethique et de Biosécurité of CIRDES and an implementing authorisation (070_2020/ADM/DG/nka) was obtained in June 2020. Infected pigs were kept in stables whose openings were protected with mosquito nets to avoid any contact with potential vectors of trypanosomes. Pigs were observed daily by a veterinarian and any animal that showed signs of distress or suffering (weight loss) during the experimental period was to be euthanised. All animals were euthanised at the end of the experiment.

### Experimental infection

The *T. b. brucei* strain MSUS/CI/2013/BE8P2P2 isolated from a pig in 2013 in the Bonon endemic HAT focus in Côte d’Ivoire was used in this experiment. The corresponding pig included in a previous study [30] was positive in the buffy coat technique (BCT) [29] and for TBR1/2 [28] and 18SF/R [9], two PCR diagnostic tests specific for *Trypanozoon*. The pig was negative in TgsGP1/2 single round PCR [34]. Isolation in the field had been performed by intraperitoneal inoculation of 0.5 mL pig whole blood, in a Naval Medical Research Institute (NMRI) mouse produced in Centre International de Recherche-Développement sur l’Élevage en zone Subhumide (CIRDES, Bobo-Dioulasso, Burkina-Faso) from paternal strains purchased from Charles River laboratories, France. The strain was kept as a cryostabilate in liquid nitrogen at CIRDES. Before the experimental infection, one stabilate was thawed and then inoculated in a mouse for parasite amplification. After amplification, harvested blood containing trypanosomes was diluted into phosphate buffered saline glucose (PSG) to achieve a concentration of 10^5^ parasites/mL in a total volume of 10 mL.

Twelve pigs 3–6 months old were acquired in a tsetse free area in western Burkina Faso. Before purchase, they had been subjected to parasitological examination using BCT, serological test using indirect-ELISA with whole pathogenic trypanosomes lysates [12], and PCR tests with primers targeting *Trypanozoon* group (primers TBR1/2), *T. vivax* (primers TVW1/2) [26], *T. congolens*e *forest* (primers TCF1/2) [25] and *T. congolense savannah* (TCS1/2) [25] to ensure that the pigs had never been in contact with trypanosomes before. At CIRDES, pigs were put in stables under mosquito nets and subsequently treated with levamisole hydrochloride (10 g per 20 L of drinking water), oxytetracycline (0.1 mL/kg of live weight), and diminazene aceturate (7 mg/kg of live weight).

Three weeks after the treatments (withdrawal period for diminazene), eight pigs randomly selected from the pool of 12 were inoculated in the left and right flank with two 500 μL intradermal injections of the suspension of 10^5^ trypanosomes/mL (infected group), i.e., 10^5^ trypanosomes. The four remaining uninfected pigs were used as the control group. The experiment lasted six months.

### Sampling and diagnostic tests

A volume of 500 μL of the 10^5^ trypanosomes/mL suspension of the strain amplified in mouse and diluted in PSG (see above) were concentrated using a mini-anion exchange centrifugation technique (mAECT) [4]. DNA was extracted from the eluate containing purified trypanosomes, using the DNeasy Blood and Tissue kit (Qiagen, Valencia, CA, USA). It was tested with TBR1/2, 18SF/R PCR and TgsGP1/2 single round PCR.

Two 70 μL capillary tubes (75 × 1.5 mm) of heparinised blood were collected from the auricular vein of each animal. These blood samples, collected the day of infection (D0), 7 days post infection (dpi) and twice a week from 7 to 189 dpi, were tested with BCT. In case of a BCT-positive result, the parasitaemia was noted following a matching method [17].

Blood samples were also collected from the jugular vein of each animal using 5 mL EDTA vacutainer^®^ tubes. After centrifugation for 15 min at 15,000 rpm, plasma samples were collected and stored at −20 °C. These plasma samples collected 19 days before infection (−19 dpi), 5 days before infection (−5 dpi), the day of infection (J0) and then weekly until 189 dpi, were used to perform TL. Plasma could not be collected at 42 dpi due to the unavailability of technicians. TL was performed using cloned populations of *T. b. gambiense* VAT LiTat 1.3, LiTat 1.5 and LiTat 1.6 as previously described [21]. For each variant, TL was considered positive if 50% to 100% of the trypanosomes were lysed.

### Statistical analysis and evaluated parameters

Statistical analyses were performed using R software version 4.0.4 (2021-02-15). The graphs were generated using the ggplot function which requires the package ggplot2 [44]. TL specificity, defined as the ability of the test to give a negative result in healthy pigs, was measured before infection on 12 pigs. TL sensitivity, defined as the ability of the test to give a positive result in infected pigs, was assessed at each sampling point on the eight infected pigs when the first antibodies became detectable. The evaluation of these two parameters and their confidence interval (CI) was performed with the “Binomial test” requiring the epiR package [38]. Fractions of positive tests in infected pigs were also estimated over the whole period, ranging from first antibody detection to the end. A non-parametric Wilcoxon test for paired data was used to assess the significance of the difference in the date of appearance of positive results for the three TL VAT. The files containing the raw data and the R script can be found in the supplementary materials.

## Results

### PCR results

The DNA extracted from purified trypanosomes of the isolated strain amplified in the mouse was positive to TBR1/2 and 18SF/R PCR but negative to TgsGP1/2 PCR.

### Parasitological investigation and infection dynamics

**Figure 1:**
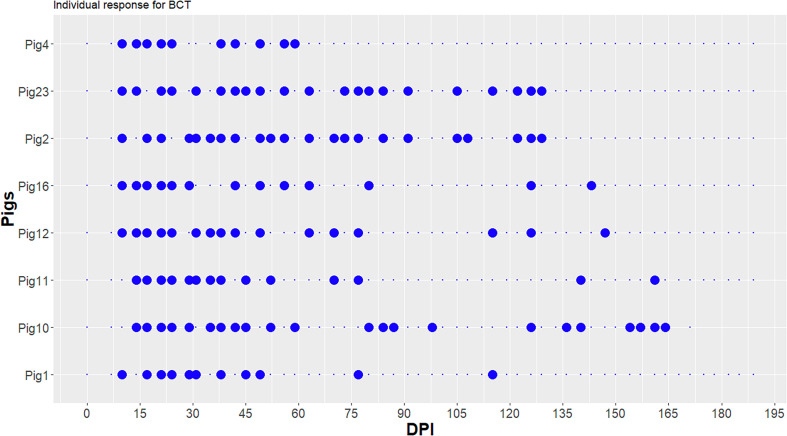
Individual buffy coat technique (BCT) results of the infected pigs during the experiment. Large blue point: BCT positive; small blue point: BCT negative; dpi: days post infection.

A total of 54 blood samples were collected on each animal, except for Pig 10 (48 blood samples) that could not be bled at 168 dpi and which was euthanised at 175 dpi due to the deterioration of its general state and weight loss. BCT was thus performed on 642 samples of which 426 were from the infected group (eight pigs) and 216 from the control group (four pigs). In the control group, no parasites were detected during the experiment. The infection dynamic for each infected pig is shown in Figure 1. All infected animals showed positive BCT several times during infection (from 10 times for pig 4 to 22 times for pig 10 and pig 2). Parasites were first detected between 10 and 14 dpi, the median time being 10 dpi. From 14 to 50 dpi, most of the pigs were positive for BCT (129/418 positive tests from 7 dpi). From 50 dpi, the BCT positivity rate began to fluctuate and decreased. Parasites were observed until a median of 136 dpi (min = 59 dpi for Pig 4, max = 164 dpi for Pig 10, interquartile range (IQR) = 126–151). Parasitaemia fluctuated and ranged from 10^5.4^ to 10^8.1^ trypanosomes/mL.

### Individual response of pigs to the trypanolysis test

**Figure 2:**
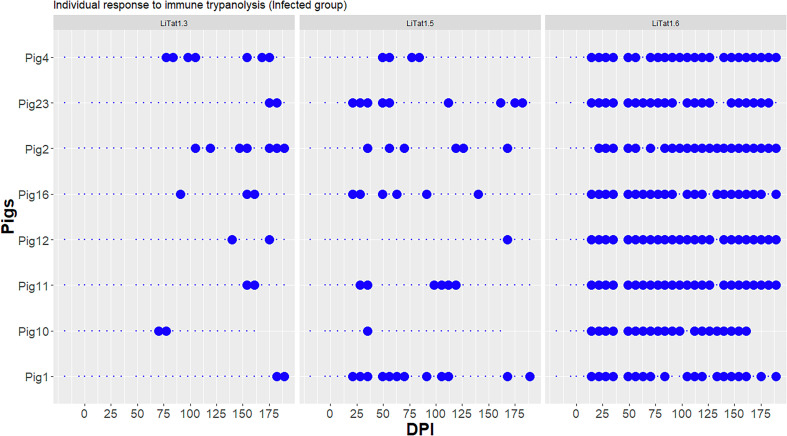
Individual trypanolysis tests (TL) results of infected pigs during the experiment. Large blue point: TL positive; small blue point: TL negative; dpi: days post infection.

A total of 29 plasma samples were collected on each pig, with two before infection, one on the infection day, and 26 during infection. Pig 23 could not be bled on the infection day and Pig 10 was euthanised at 175 dpi. A total of 343 samples were analysed in TL with the three VATs, 227 from the infected group (8 pigs) and 116 from the control group (4 pigs). All pigs before infection and at 0 dpi, as well as all control pigs during the experiment, were negative in TL. The individual TL responses of the infected pigs for the three VATs are shown in Figure 2. Trypanolysis with the three VATs was positive at least once in all infected pigs, but the dpi of the first positivity and the positivity rate varied between the three VATs. The first positive animals were observed at 14 dpi for LiTat 1.6 (7 positive pigs at this time point), 21 dpi for LiTat 1.5 (3 pigs) and 70 dpi for LiTat 1.3 (1 pig). For LiTat 1.6, the median time to become positive was 14 dpi (IQR 14–14, maximum 21 DPI), for LiTat 1.5, it was 32 dpi (IQR 21–39, maximum 168 dpi) and for LiTat 1.3, the median time for positivity was 123 dpi (IQR 88–159, maximum 182). The times of first positivity were significantly different between LiTat 1.5 and LiTat 1.3 (*p*-value = 0.02), between LiTat 1.6 and LiTat 1.3 (*p*-value = 0.007), and between LiTat 1.6 and LiTat 1.5 (*p*-value = 0.01).

Evolution of the response to the three VATs showed that the positive responses to LiTat 1.6 persisted over time and were homogeneous for the eight pigs (only 21 samples negative/196 samples tested from 14 dpi). The pigs were positive in 72% (18/25 for Pig 1) to 92% (23/25 for Pigs 11 and 12) of the time points. On the contrary, there was a very high variability for VATs LiTat 1.3 and LiTat 1.5, whose positivity was more sporadic during the experiment. From 14 dpi, the pigs tested positive 4% (Pigs 10 and 12) to 48% (Pig 1) of the time points for LiTat 1.5 and 8% (Pigs 1, 11, 12 and 23) to 28% (Pigs 2 and 4) of time points for LiTat 1.3.

### Sensitivity and specificity of the trypanolysis test

**Figure 3:**
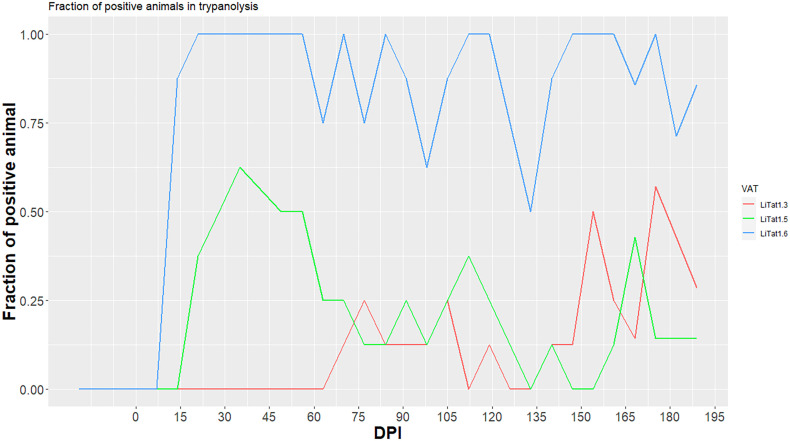
Frequencies of trypanolysis test (TL) positive results in infected pigs during the experiment. Lines indicate the fraction of TL positive pigs (red for LiTat 1.3, green for LiTat 1.5, blue for LiTat 1.6); dpi: days post infection; VAT: variant antigen type.

TL showed specificity of 100% (95% CI = 74–100, *n* = 12 pigs), for the three VATs. In addition, all tests (116) in the control group were negative. Frequencies of TL positive results in infected pigs during the experiment are shown in Figure 3. Sensitivity of TL on eight infected pigs for the three VATs varied throughout the experimental period from the first detection of antibodies, i.e., 14 dpi.

LiTat 1.6 TL had the highest sensitivity ranging from 50% (95% CI = 16–84%) to 100% (95% CI = 63–100%), the fraction of positive tests being 89% (95% CI = 84–93%) over the whole period. For LiTat 1.3 TL, sensitivity ranged from 0% (95% CI = 0–37%) to 57% (95% CI = 18–90%), the fraction of positive tests being 14% (95% CI = 9–19%). For LiTat 1.5 TL, sensitivity ranged from 0% (95% CI = 0–37%) to 63% (95% CI = 24–91%), the fraction of positive tests being 23% (95% CI = 17–29%).

## Discussion

An animal reservoir for *T. b. gambiense* is one of the factors that could compromise the goal of interrupting the transmission of g-HAT by 2030 [3, 13]. Thanks to its performance in terms of specificity and sensitivity described in humans, TL has become the reference immunological diagnostic tool for the surveillance of g-HAT [3, 7, 23]. However, its reliability as a diagnostic tool of *T. b. gambiense* infection in animals has been questioned and further experimental studies proposed to confirm or reject the *T. b. gambiense* specificity of this method [39]. This was the main objective of this experimental infection conducted in pigs. Pigs are known to often carry multiple trypanosome infections and are suspected to be a *T. b. gambiense* reservoir in g-HAT areas, particularly in Côte d’Ivoire [20, 30, 39] and Cameroon [31, 32, 36].

For this experimental infection, we used a strain isolated from a pig potentially infected by several strains of different trypanosome species. We therefore cannot completely exclude that the strain used is a mix of several ones. Not using a clone is a limitation of the study. However, the most important aspect was to rule out the presence of *T. b. gambiense*. The results of the diagnostic tests performed on the corresponding pig in 2013 [30] as well as the TgsGP PCR-negative result obtained on the DNA extracted from the purified strain pointed in this direction. MSUS/CI/2013/BE8P2P2 was also characterised as a single *T. b. brucei* strain by microsatellite markers (data not shown).

The BCT results show that all inoculated pigs became infected. The dynamic of infection was homogenous in the eight pigs with a prepatent period between 10 and 14 dpi in the range of what was already observed in other experimental trypanosome infections for pigs [18, 33]. Concerning TL, our results confirmed the high diagnostic specificity of the test performed with the three VATs to uninfected pigs. Our study also revealed that all infected pigs developed antibodies directed against the three VATs used for TL, but with different patterns of results.

There was a significant difference in first detection of positivity to the three VATs. In all infected pigs, antibodies against LiTat 1.6 were the first to be detected after two weeks of infection. One week later, anti-LiTat 1.5 antibodies were detected and about two months later, anti-LiTat 1.3 antibodies appeared. The sequential appearance of VATs with a dominant VAT in the early stage of infection, i.e., in the first weeks of infection, is a characteristic of African trypanosomes [5, 15, 37, 39]. LiTat 1.3, LiTat 1.5 and LiTat 1.6 have been described as dominant VATs in early *T. b. gambiense* infection [41]. Also RoTat 1.2 is the dominant VAT of *T. evansi* [42]. In the present experiment, only anti-LiTat 1.6 antibodies appeared early and were sustainably produced, indirectly suggesting that LiTat 1.6 is a dominant VAT in *T. b. brucei* in which it has already been described [41].

We also observed that anti-LiTat 1.6 antibody detection by TL was persistent over time in all pigs, whereas detection of anti-LiTat 1.3 and LiTat 1.5 antibodies was rather sporadic during the experiment. This fluctuating detection of anti-LiTat 1.3 and 1.5 antibodies was unexpected and is different from results observed in humans infected by *T. b. gambiense* for which trypanolysis remains positive during infection and even for several years after treatment [19, 22]. Considering the hypothesis that VATs LiTat 1.3 and LiTat 1.5 can be expressed by a *T. b. brucei* strain, this sporadic detection could be associated with a very low level of antibodies specific to these VATs (at concentrations below the detection threshold of the TL), low reactivity or unknown immunological mechanisms involving sporadic production of these antibodies. According to some authors, the level of specific antibodies is higher for early variants that appear a few weeks after infection, which would be the case for LiTat 1.6 in the present study, and the antibody level decreases with variants that appear later [15, 35]. The lack of sensitivity of LiTat 1.3 and LiTat 1.5 TL could be explained by the fact that these variants are expressed at very low levels by *T. b. brucei.* Similar results of poor sensitivity using TL for g-HAT on samples from cattle were observed in an AAT endemic region in Uganda [27]. Another hypothesis is that some *T. b. brucei* VATs have antigenic similarities (similar epitopes) with LiTat 1.3 and 1.5 ones and cause cross-reactions.

Nevertheless, our study provides the first experimentally acquired evidence that LiTat 1.3 and LiTat 1.5 TL are not specific of *T. b. gambiense* and are thus unsuitable tools to unequivocally identify a potential animal reservoir of *T. b. gambiense*. To address the crucial question of the presence of an animal reservoir [39] in the framework of the elimination of g-HAT transmission, new reliable and specific tools for the identification of *T. b. gambiense* in animals will thus be required. This article does not call into question the specificity of TL in humans as already evidenced [8]. In fact, humans who can temporarily be infected with *T. b. brucei* are resistant to this parasite thanks to Trypanosome lytic factor-1 in the blood [16], and we may assume that no antibodies are produced, thus preventing TL from detecting such infections. Our study also suggests that the LiTat 1.6 TL could be used as a serological test for the diagnosis of *T. brucei* s.l. infections in pigs, but without being able to differentiate between *T. b. brucei* and *T. b. gambiense*.

## Supplementary materials

The Supplementary materials of this article are available at https://www.parasite-journal.org/10.1051/parasite/2022063/olm. *Supplementary data1*: Trypanolysis data in R analysis format.
*Supplementary data2*: Buffy coat technique (BCT) data in R analysis format.
*Supplementary data3*: First detection of parasite.
*Supplementary data4*: Script with details of the data analysis.
*Supplementary data5*: Buffy coat technique (BCT) and trypanolysis raw data.
